# Association between Metabolic Syndrome Components and Cardiac Autonomic Modulation among Children and Adolescents: A Systematic Review and Meta-Analysis

**DOI:** 10.3390/biology10080699

**Published:** 2021-07-22

**Authors:** Rashmi Supriya, Fei-Fei Li, Yi-De Yang, Wei Liang, Julien S. Baker

**Affiliations:** 1 Centre for Health and Exercise Science Research, Department of Sport, Physical Education and Health, Hong Kong Baptist University, Kowloon Tong 999077, Hong Kong; lifeifei@hkbu.edu.hk (F.-F.L.); wliang1020@hkbu.edu.hk (W.L.); 2 School of Medicine, Hunan Normal University, Changsha 410081, China; yangyide2007@126.com

**Keywords:** metabolic syndrome, cardiac autonomic modulation, heart rate variability, children, adolescents, pre-pubertal adolescents, meta-analysis, systematic review

## Abstract

**Simple Summary:**

The clustering of metabolic syndrome (MetS) risk factors is becoming more prevalent in young people (up to the age of 19 years) leading to the development of type 2 diabetes (T2D) and cardiovascular diseases in early adulthood. The impact of MetS risk factors on cardiac autonomic modulation (CAM) or vice versa have been noted to track from childhood to pre-adolescence and adolescence. Understating associations in this age group may help improve the clinical outcomes of the MetS, even when MetS symptoms are not visible. Potential damage from each individual MetS component and the ability to predict early cardiac damage or upcoming cardiovascular events is very important. Therefore, the present systematic review and meta-analysis investigated the associations between CAM and MetS risk factors individually to verify which MetS risk components were significantly correlated with which heart rate variability (HRV) indices before or at the onset of the MetS among young people. The purpose of this review was to outline the importance of potentially screening HRV indices in young people even with only one MetS risk factor, as a pre-indicator for early cardiovascular risk stratification. Cross-sectional studies that examined the relationship of MetS risk factors with HRV indices were searched using four databases including PubMed, the Cochrane clinical trials library, Medline and the Web of Science. Correlation coefficients with 95% confidence intervals (95% CI), and random effects meta-analyses of the association between MetS risk factors with HRV indices were performed. Our results propose that lipid profiles including high density lipoprotein (HDL) and triglycerides (TGs), waist circumference (WC) and blood pressure (BP) are associated with CAM in young people up to the age of 19 years. The use of HRV indices to predict future MetS risk, and relationships with individual risk factors including HDL, BP, WC and TGs, were established. Furthermore, arterial pressure, respiration, stress and physical activity must be taken into consideration for future studies along with CAM related to young people (up to the age of 19 years), and it is recommended to explore further the associations reported here, as CAM is not the only determinant of neurovisceral regulation.

**Abstract:**

Background: the clustering of metabolic syndrome (MetS) risk factors is becoming more prevalent in children, leading to the development of type 2 diabetes (T2D) and cardiovascular diseases in early adulthood. The impact of MetS risk factors on cardiac autonomic modulation (CAM) or vice versa has been noted to track from childhood to pre-adolescence and adolescence. Understating associations in this age group may help to improve the clinical outcomes of the MetS, even when MetS symptoms are not visible. Potential damage from each individual MetS component and the ability to predict early cardiac damage or upcoming cardiovascular events is very important. Therefore, the present systematic review and meta-analysis investigated the associations between CAM and MetS risk factors individually to verify which of the MetS risk components were significantly correlated with heart rate variability (HRV) indices before or at the onset of the MetS among young people. The purpose of this review was to outline the importance of potentially screening HRV indices in young people even with only one MetS risk factor, as a pre-indicator for early cardiovascular risk stratification. Methods: cross-sectional studies that examined the relationship of MetS risk factors with HRV indices were searched using four databases including PubMed, the Cochrane clinical trials library, Medline and the Web of Science. Correlation coefficients with 95% confidence intervals (95% CI), and random effects meta-analyses of the association between MetS risk factors with HRV indices were performed. Results: out of 14 cross-sectional studies and one case-control study, 8 studies (10 data sets) provided association data for the meta-analysis. Our results indicated significant positive correlations for systolic blood pressure (SBP) (correlation coefficient 0.13 (95%CI: 0.06; 0.19), I^2^ = 47.26%) and diastolic blood pressure (DBP) (correlation coefficient 0.09 (95%CI: −0.01; 0.18), I^2^ = 0%) with a Low Frequency/High Frequency ratio (LF/HF). Significant negative correlations for waist circumference (WC) (correlation coefficient −0.12 (95%CI: −0.19; −0.04), I^2^ = 51.50%), Triglycerides (TGs) (correlation coefficient −0.09 (95%CI: −0.15; −0.02), I^2^ = 0%) and ≥2 MetS risk factors (correlation coefficient −0.10 (95%CI: −0.16; −0.03), I^2^ = 0%); with high frequency (HF) were revealed. Significant positive correlations for high density lipoprotein (HDL) (correlation coefficient 0.08 (95%CI: 0.05; 0.11), I^2^ = 0%) and significant negative correlations of ≥2 MetS risk (correlation coefficient −0.04 (95%CI: −0.12; 0.03), I^2^ = 0.0%) with low frequency (LF) were revealed. Significant negative correlations for TGs (correlation coefficient −0.09 (95%CI: −0.23; 0.05), I^2^ = 2.01%) with a mean square root of the sum of differences between mean time between two successive intervals (rMSSD) and significant positive correlation of HDL (correlation coefficient 0.09 (95%CI: −0.01; 0.19), I^2^ = 0.33%) with standard deviation of the time between two successive intervals (SDNN) were also revealed. An Egger’s test indicated that there was no obvious publication bias for any of the above relationships except for TGs and rMSSD. The significance level stipulated for the meta-analysis was *p* < 0.05. Conclusions: lipid profiles (HDL and TGs), WC and BP were associated with CAM in young people up to the age of 19 years. The use of HRV indices to predict future MetS risk, and relationships with individual risk factors including HDL, BP, WC and TGs, were established. Future studies related to young people (up to the age of 19 years) are recommended to explore the associations reported here further.

## 1. Introduction

The cardiovascular system is regulated mainly by the autonomic nervous system [1]. Cardiac autonomic modulation (CAM) is assessed by heart rate variability (HRV) and has been considered as a potent marker of cardiovascular risk [2,3]. HRV is defined as the variability between consecutive heart beats. It is assessed by analyzing heart beat intervals using time and frequency domain techniques [4]. Time domain measures have been described as the heart rate at any point in time or the measure of intervals between successive normal complexes. Frequency domain analysis describes the periodic oscillations of the heart rate signals.

Low HRV indicates that there is a reduction in parasympathetic cardiac control. This has been associated with type 2 diabetes (T2D), with traditional/Metabolic syndrome (MetS) and non-traditional cardiovascular risk factors (stress, cortisol level, inflammatory biomarkers) [5,6]. MetS or pre-diabetes is the clustering of metabolic risk factors (CMRF) including high blood pressure (BP), high triglyceride (TGs) levels, low/high density lipoprotein (HDL), high fasting glucose levels (FGL), high waist circumference (WC) and high/low density lipoprotein (LDL) [7]. The signs and symptoms of T2D appear suddenly, and MetS assessment is a good method to detect people at risk. MetS is defined as a cluster of three or more risk factors [8], and it requires invasive techniques utilizing blood withdrawal and analysis. However, in young people (children, pre-pubertal adolescents and adolescents), the constellation is difficult to define, leading to unclear diagnosis for clinical care [7]. Focusing attention on young people with CMRF should be emphasized over the requirement to define a pediatric MetS condition.

The question arises whether there is potential for early detection of T2D or cardiovascular diseases prior to the signs and symptoms of MetS becoming apparent in young people. Micro-vascular analysis of HRV may be a good prior indicator of future cardiovascular risk [9,10,11]. CAM monitoring is a non-invasive and is a relatively cheap method of analysis. During growth at a young age, hormonal fluctuations promote the growth of lean fat and bone mass, making youth more prone to insulin resistance [12]. The young people are in their sensitive period of physiological and psychological development, which means they are not able to cope with learning stress, sleep deprivation, interpersonal tension, and other external pressures and problems, thus they are more likely to become depressed [13,14]; this may be a contributory factor for future cardiovascular risk. However, it is not well established as an indicator of cardiovascular disease in children and adolescents who are at high risk of T2D and related cardiovascular disorders. Early information about the change in cardiac autonomic control may help predict upcoming CMRF before the MetS develops and shows obvious signs and symptoms among young people.

The relationship between CAM or low HRV and compromised cardiovascular health has been comprehensively studied in adults; however, such studies are scarce in adolescents and children. In a recent meta-analysis comprising millions of subjects, Nanayankkara et al. [15] observed that younger rather than older age people with diabetes were associated with a higher risk of mortality and vascular disease. They also proposed that identification and quantification of the increased risk of mortality and vascular disease conferred by a younger age at T2D diagnosis may enable risk stratification of individuals in the early stages of the condition. This would provide greater opportunities for interventions to reduce the risk of complications, associated morbidity and mortality in this population for developing T2D.

Based on this assumption, some studies have demonstrated correlations between CAM and cardiovascular risk factors early in childhood/adolescence in order to discriminate between young people at increased risk of cardiovascular diseases. Farah et al. in a cross sectional study including 1152 adolescent boys demonstrated that lower HRV measures were associated with CMRF [16]. Rodriguez-Colon et al. [17] also demonstrated the relationship between MetS and CAM in a Penn State Children cohort study of 421 adolescents, and concluded that all MetS components except HDL were associated significantly with reduced HRV. They suggested that CAM could be a predictor of future pre-diabetes in apparently healthy adolescents [17]. Biljon et al. [18] demonstrated that HRV was significantly correlated with HDL levels in 34 South African children [18]. In a Panic Study by Leppänen et al. [19], associations between CMRF with HRV revealed a higher cardio metabolic risk score, and BP and insulin levels were associated with low HRV. They also proposed that monitoring CAM may improve the clinical management of MetS and cardiovascular diseases from an early age [19]. Conversely, Zhou et al. [20] demonstrated that only obesity and elevated systolic BP (SBP) were associated with reduced HRV in 180 Chinese children [20]. Considerable research has focused on these parameters and this systematic review and meta-analysis attempts to compile studies that have observed this concept among children, pre-pubertal populations and adolescents separately. The impact of MetS risk factors on cardiac autonomic nervous system regulation has been noted to track from childhood into pre-adolescence into adolescence, and understating the cumulative impact of their association on this young age group will help to improve the clinical management of MetS even when symptoms are not visible.

Most of the studies examined did not consider maturation variables, which affects the relationship between HRV and individual MetS risk factors. This may be a reason for the variation in results among the associations of HRV indices with MetS risk factors. For instance, significant positive associations between HDL and standard deviation of the time between two successive intervals (SDNN) were reported by van Biljon et al. and Rodriguez-Colon et al. [17,18]. Contrary to these findings, Leppänen et al. [19] and Zhou et al. reported that there were no significant associations between HDL and SDNN. Therefore, this review considered young people up to the age of 19 years as candidates for MetS in order to avoid the confounding effects of maturity status among the population. Cardiovascular disease is rising at an alarming rate in youth, and prior identification of vulnerability is required urgently. Also, there are several studies that show that cardiovascular risk factors are associated with HRV when MetS has been identified in young people [17,19]. Potential damage from each individual MetS component needs to be avoided, and the ability to predict early cardiac damage or upcoming cardiovascular events is very important.

Therefore, the present study comprises a systematic review and meta-analysis investigating associations between CAM and MetS risk factors individually. This was done to verify which cardiovascular risk factors are significantly correlated with HRV indices prior to or during the onset of the MetS in young people.

## 2. Methodology

This systematic review was conducted and reported in accordance with the Preferred Reporting Items for Systematic Reviews and Meta-Analyses (PRISMA) statement [19]. The study has been registered in PROSPERO (CRD42021247766).

### 2.1. Search Strategy

The search timeline included studies published until March 2021 and was performed by two researchers using four databases including PubMed, the Cochrane clinical trials library, Medline and the Web of Science. The titles/abstract of the articles were searched using “autonomic nervous system OR cardiac autonomic neuropathy OR heart rate variability OR sympathetic nervous system OR parasympathetic nervous system” and “metabolic syndrome OR pre-diabetes OR MetS OR MetX OR cardiovascular risk factors OR cardio-metabolic risk factors” and “adolescents OR children OR youth OR teenagers OR young people OR students OR primary school students OR secondary school students” as keywords. The publication duration was from the beginning of the scientific platform until March 2021, and language was limited to English.

### 2.2. Exclusion and Inclusion Criteria

All articles were recovered using the keywords and were screened for title and abstract to assess their eligibility. Inclusion criteria for the selection of studies included (1) the study should be a relationship study, (2) subjects should be children or adolescents (age not more than 19 years), (3) should contain measurement of at least one HRV index, (4) should have measurements of at least two or more cardio-metabolic risk factors (Body Mass index (BMI), WC, BP, TGs, HDL, FGL and LDL). Subjects with diabetes or cardiovascular diseases and neurological or mental disabilities were excluded.

### 2.3. Quality Assessment

Methodological quality and risk of bias of studies was confirmed using the Joanna Briggs Institute Critical Appraisal Checklist for Prevalence Studies (JBI) [21], from the University of Adelaide, Australia (JBI, 2016). This consists of nine questions facilitating the final methodological quality score. From 0 to 6 is classified as moderate or low quality and from 7 to 9 is classified as high quality. The classification indices were based on the work of Kasten et al. [22].

### 2.4. Data Extraction and Analysis

Two authors, RS and FL, extracted data independently. The extracted data included the study characteristics e.g., title, author, publication year, country, sample size, sex, age of participants, BMI, SBP, diastolic BP (DBP), WC, TGs, LDL, HDL, FGL, ≥2 MetS risk factors, mean square root of the sum of differences between mean time between two successive intervals (rMSSD), Low Frequency (LF), High Frequency (HF), Low Frequency/High Frequency ratio (LF/HF), SDNN and HRV Z score. We selected time domain and frequency domain HRV parameters. SDNN and rMSSD were the indices selected under the time domain, and LF, HF and LF/HF were selected from the frequency domain HRV indices. The clinical meanings of the HRV indices used have been presented in the synoptic table in the Supplementary Materials (Table S1). Also, BMI, SBP, DBP, WC, TGs, LDL, HDL, FGL, ≥2 MetS risk were selected from MetS risk factor indices for meta-analysis. We extracted the beta coefficient (β) and correlation coefficient (r) between MetS risk factors and HRV indices. Finally, β was converted to r based on the published formula [23]. We further used these HRV indices and MetS risk factors for concluding overall variability, parasympathetic activity, baroreflex activity and sympathovagal balance.

Meta-essential 1.5 software (Copenhagen: The Nordic Cochrane Centre) [24] was used for the data analysis, and a random-effects analysis was performed on data collected from the selected studies. Forest plots were generated and the significant correlated HRV index whose heterogeneity was ≤50% was considered significant in this study. The HRV indices that were reported by less than three studies on data sets were not included for the meta-analysis. HRV indices that were reported by ≥3 studies were included in our meta-analysis. In order to measure effect size, the global Z effect size test was applied, where a 95% confidence interval was used. The Egger’s regression intercept and funnel plot were used to examine publication bias [24]. The I^2^ tests were used to analyze the homogeneity of the studies. Since we did not find more than six data sets for any of our parameters selected, we did not perform subgroup analysis for our significant relationships with heterogeneity, and we preferred to present the real values of the outcome measures. Only significant results with a low heterogeneity of ≤50% were considered important for discussion in this study; however, we have reported the heterogeneously significant results in the result section. For the meta-analysis, the following variables were used: rMSSD, HF, and LF, LF/HF, SDNN, SBP, DBP, TGs, HDL, FGL, BMI, WC and ≥2 MetS risk factors. The significance level stipulated for the meta-analysis was *p* < 0.05.

## 3. Results

**Figure 1:**
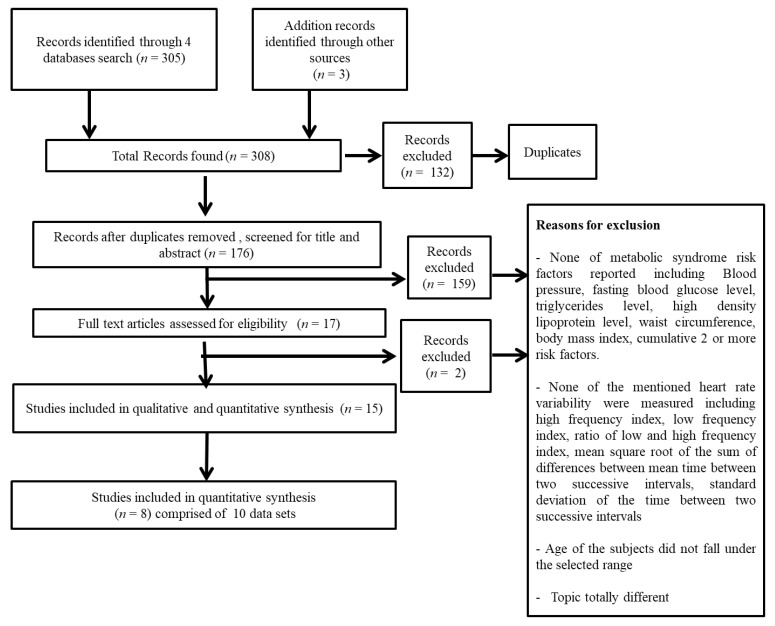
Flowchart of the selection of studies for the systematic review and meta-analysis (2 data sets taken from Leppänen et al. 2020 and 2 data sets from van Biljon et al. 2019.

Initially, 308 articles from 4 databases including PubMed (*n* = 39), Medline (*n* = 96), Web of science (*n* = 117), and Cochrane clinical trials (*n* = 53) were found and 176 were considered for inclusion after removing duplicates. Following screening of 176 articles, 17 articles were considered eligible for full text assessment that met the pre-established criteria (Figure 1). Finally, 15 studies were included for qualitative and quantitative synthesis. Only eight studies were association studies and were eligible for meta-analysis, in which two studies had two data sets. Two data sets were extracted from Leppänen et al. 2020 and van Biljon et al. 2019. Therefore, in this meta-analysis, 10 data sets were used.

Studies were assessed for the evaluation of methodological quality and risk of bias, and the “Jonna Briggs” Institute critical appraisal checklist for prevalence studies was applied. All articles selected scored within the stipulated range of high methodological quality and risk of bias (7 to 9) Supplementary Materials (Table S2).

Summary of the studies selected for this systematic and meta-analysis are presented and are available in the Supplementary Materials (Table S3). Fifteen studies were published between 2009 to 2021. Among these, only eight studies were association studies and were published between 2012 to 2020. Regions of publications were from South America (*n* = 5), all from Brazil [16,25,26,27,28]; North America (*n* = 2), including Pennsylvania (*n* = 1) [17] and Tennessee (*n* = 1) [29]; Asia (*n* = 1), including only Mainland China (*n* = 1) [20]; Europe (*n* = 5) including Finland (*n* = 1) [19], Greece (*n* = 1) [30], Spain (*n* = 1) [31] and Holland (*n* = 1) [32], and from Africa (*n* = 1), including only South Africa [18]. Sample size per association study ranged from 39 to 1152, with a total of 3745 subjects. Among 6767 subjects, 5120 were boys that comprised 75% of the total subjects. Also, total subjects taken in the meta-analysis were 3745. Among 3745 subjects, 3035 were boys, that comprised 81% of the total population. The mean age ranged from 5.6 ± 0.4 to 16.9 ± 2.24 years.

### 3.1. SBP Association with CAM

For SBP, five studies showed significant negative associations with HF [16,19,20,27,28]. Three studies showed significant negative associations with SDNN and rMSSD [16,19,20]. Three studies showed significant positive associations with the LF/HF ratio [6,16,20]. On the other hand, two studies [16,27] showed significant positive and two studies [19,20] reported negative significant associations with LF.

**Figure 2:**
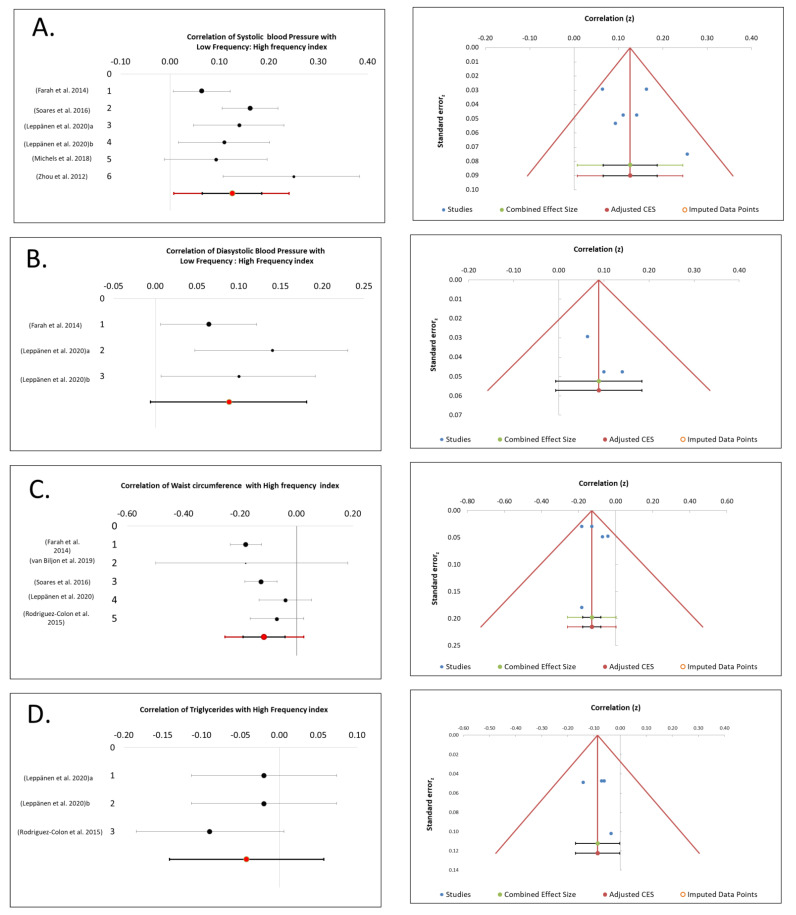

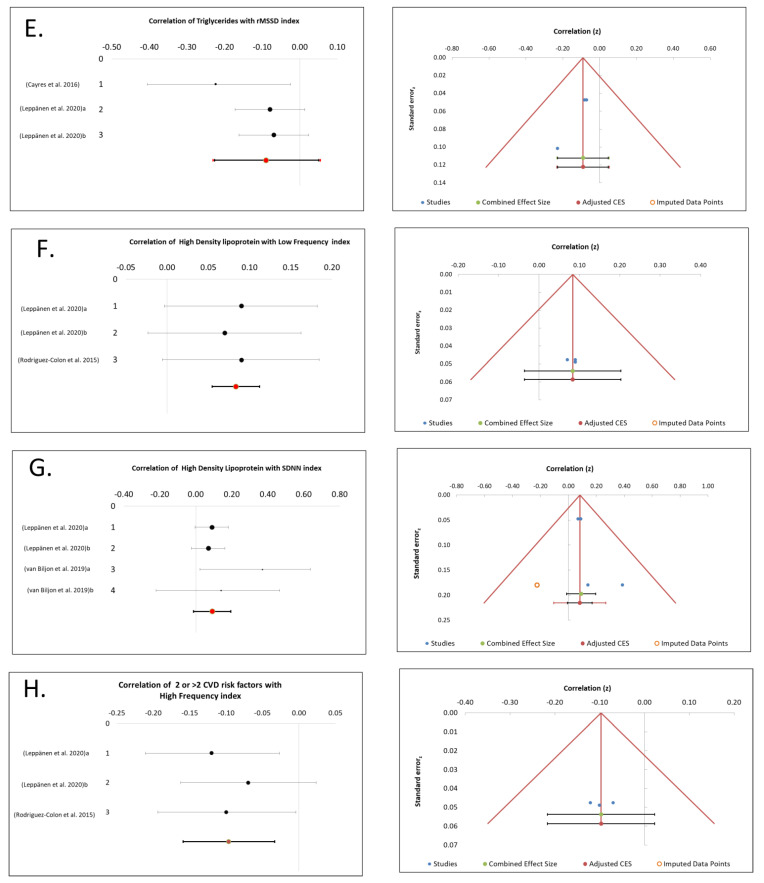

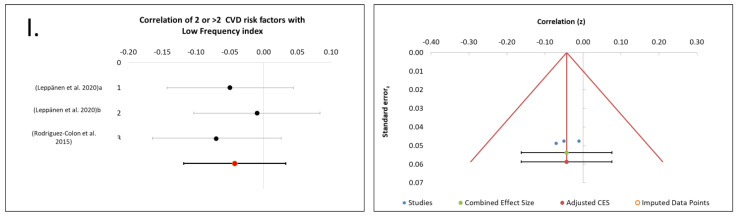
(**A**–**I**) are Forest plots and Funnel plots of pooled significant correlations between cardio metabolic risk factors and heart rate variabilty indices along with its publication bias, respectively including (**A**). (**A**). Correlation of systolic blood pressure with low frequency: high frequency index; (**B**). Correlation of diastolic blood pressure with low frequency: high frequency index; (**C**). Correlation of waist circumference with high frequency index; (**D**). Correlation of Triglycerides with high frequency index; (**E**). Correlation of triglycerides with rMSSD index; (**F**). Correlation of high density lipoprotein with Low frequency index; **G**. Correlation of high density lipoprotein with SDNN index; (**H**). Correlation of 2 or >2 CVD risk factors with high frequency index; (**I**). Correlation of 2 or >2 CVD risk factors with low frequency index. All the correlations charts are accompanied by their Eggers test graph, rMSSD is mean square root of the sum of differences between mean time between two successive intervals, SDNN is standard deviation of the time between two successive intervals and CVD is cardio-vascular disease.

For SBP, our results indicate LF/HF was only significantly correlated without heterogeneity with six datasets and 3723 subjects analyzed (Z = 5.30, *p* < 0.00001, correlation coefficient 0.13 (95%CI: 0.06; 0.19), I^2^ = 47.26%) (Figure 2A). An Egger’s test (t = 1.26, *p* = 0.27) indicated that there was no significant publication bias for this relationship (Figure 2A). Also, HF and rMSSD were found to be significantly correlated but with high heterogeneity. HF with five datasets and 3289 subjects was analyzed (Z = −5.03, (*p* < 0.00001), correlation coefficient −0.15 (95%CI: −0.24; 0.07), I^2^ = 65.03%). rMSSD with four datasets and 2137 subjects was analyzed (Z = −2.25, (*p* = 0.025), correlation coefficient −0.20 (95%CI: −0.45; 0.08), I^2^ = 95.54%).

### 3.2. DBP Association with CAM

For DBP, two studies reported significant negative associations with SDNN and rMSSD [16,19]. Only one study reported significant negative associations with HF. Also, only one study reported significant positive associations with LF/HF and LF [16].

For DBP, our results indicate that the LF/HF variable was only found significantly correlated without heterogeneity with a total number of three datasets and 2038 subjects analyzed (Z = 4.04, (*p* < 0.00001), correlation coefficient 0.09 (95%CI: −0.01; 0.18), I^2^ = 0%) (Figure 2B). An Egger’s test (t = 2.10, *p* = 0.28) indicated that there was no significant publication bias for this relationship (Figure 2B). Also, SDNN was significantly correlated but with high heterogeneity with three datasets and 2038 subjects analyzed (Z = −1.96, (*p* = 0.05), correlation coefficient −0.18 (95%CI: −0.51; 0.21), I^2^ = 94.48%).

### 3.3. WC Association with CAM

For WC, four studies reported significant positive associations with LF/HF [16,17,18,27]. Three studies [17,18,27] reported significant negative associations and one study [16] reported a positive association with HF. Two studies [17,18] reported significant negative associations and one study [16] reported a significant positive association with rMSSD. Also, one study [17] reported significant negative associations and one study [16] reported a significant positive association with SDNN. Plaza-Florido et al. reported that WC was negatively associated with a cumulative HRV score. On the contrary, a HRV score has been shown to have no impact on high BP, pre-diabetes in overweight obese youth by Lee et al.

For WC, we found that HF was only found significantly correlated without heterogeneity with five datasets and 3202 subjects analyzed (Z = −4.29, (*p* < 0.00001), correlation coefficient −0.12 (95%CI: −0.19; −0.04), I^2^ = 51.50%) (Figure 2C). An Egger’s test (t = 0.63, *p* = 0.57) indicated that there was no significant publication bias for this relationship (Figure 2C). Also, LF/HF was found significantly correlated but with heterogeneity; six datasets and 3555 subjects were analyzed (Z = 1.98, (*p* = 0.047), correlation coefficient 0.07 (95%CI: −0.02; 0.15), I^2^ = 62.41%).

### 3.4. TGs Association with CAM

For TGs, three studies reported significant negative associations with both HF and rMSSD [17,19,28]. Two studies reported significant negative associations with both LF and SDNN [17,19]. Two studies [17,19] reported a significant positive association and one study [6] reported a significant negative association with LF/HF.

For TGs, our results indicated that HF and rMSSD were found significantly correlated with no heterogeneity. HF with four datasets and 1406 subjects was analyzed (Z = −3.99, (*p* < 0.00001), correlation coefficient −0.09 (95%CI: −0.15; −0.02), I^2^ = 0%) (Figure 2D). An Egger’s test (t = 0.51, *p* = 0.66) indicated that there was no obvious publication bias for this relationship (Figure 2D). rMSSD with three datasets and 985 subjects was analyzed (Z = −2.77 (*p* = 0.006), correlation coefficient −0.09 (95%CI: −0.23; 0.05), I^2^ = 2.01%) (Figure 2E). An Egger’s test (t = −9.53, *p* = 0.07) indicated that there was publication bias for this relationship (Figure 2E).

### 3.5. HDL Association with CAM

For HDL, three studies [17,18,19] reported significant positive associations and one study [28] reported a significant negative association with HF. Two studies [18,19] reported significant positive associations and one study [28] reported a significant negative association with rMSSD. Two studies reported significant positive associations with SDNN [18,19]. Two studies [6,17] reported significant positive associations and one study [19] reported a significant negative association with LF/HF [18,19]. Two studies reported significant positive associations with LF [17,19]. Consistently, Plaza-Florido et al. reported that HDL was positively correlated with a HRV Z score. 

For HDL, we found that LF and SDNN were significantly correlated without heterogeneity. LF with three data sets and 1307 subjects was analyzed (Z = 12.38, (*p* < 0.00001), correlation coefficient 0.08 (95%CI: 0.05; 0.11), I^2^ = 0%) (Figure 2F). An Egger’s test (t = 0.57, *p* = 0.67) indicated that there was no significant publication bias for this relationship (Figure 2F). SDNN with four datasets and 954 subjects was analyzed (Z = 2.82, (*p* = 0.005), correlation coefficient 0.09 (95%CI: −0.01; 0.19), I^2^ = 0.33%) (Figure 2G). An Egger’s test (t = 1.95, *p* = 0.19) indicated that there was no obvious publication bias for this relationship (Figure 2G).

### 3.6. LDL Association with CAM

For LDL, one study reported that there was no correlation with any of the HRV indices [28]. However, both adjusted data sets from one study reported a significant positive association and a significant negative association with HF and LF, respectively [18]. We did not find any significant correlation between LDL and any of the HRV indices.

### 3.7. BMI Association with CAM

For BMI, three studies reported significant negative associations with HF [16,18,27]. Two studies reported significant negative associations with rMSSD [16,18]. Two studies reported significant positive associations with LF/HF [16,27]. One study [16] reported a significant positive and one study [27] reported a significant negative association with LF. Only one study reported a significant negative association with SDNN [16]. We did not find any significant correlation between BMI and any of the HRV indices.

### 3.8. FGL Association with CAM

For FGL, three studies unanimously reported that there was no relationship between FGL and HRV indices [19,29,30]. We had two data sets only for all HRV indices. Therefore, we did not perform any quantitative analyses.

### 3.9. Cluster of ≥2 MetS Risk Factors Association with CAM

Farah et al. aimed to establish cutoffs of HRV parameters for examining their association with cardiovascular risk factors in adolescents from Brazil. Their binary logistic regression analysis showed that HRV indices including SDNN, rMSSD, LF, HF were independently associated with the clustering of MetS risk factors (sum of abdominal obesity, high blood pressure, overweight and low physical activity level) [26]. They proposed that HRV cutoff points have moderate to high sensitivity in predicting cardiovascular risk and proposed that HRV frequency are a better domain than time domain indices. Plaza-Florido et al. studied the impact of different Kubios threshold-based artifact correction levels on the relationship between MetS risk factors and HRV scores, to explain the inconsistencies in results of associations between MetS risk factors and HRV. They reported that only WC and HRV scores were negatively associated with HRV scores using medium and strong kubios filters in children (β = −0.22 and −0.24, *p* = 0.03 and 0.02, respectively). They also included heart rate as a covariate, especially in children and adults. They reported that most associations disappeared after including heart rate as a covariate. Zhou et al. have reported that as the number of MetS risk factors increases, the HRV decreases.

For ≥2 MetS risk factors, we found that HF and LF were significantly correlated with no heterogeneity. HF with three data sets and 1307 subjects were analyzed (Z = −6.56, (*p* < 0.00001), correlation coefficient −0.10 (95%CI: −0.16; −0.03), I^2^ = 0%) (Figure 2H). An Egger’s test (t = −0.11, *p* = 0.93) indicated that there was no obvious publication bias for this relationship (Figure 2H). LF with three data sets and 1307 number of subjects were analyzed [Z = −2.43 (*p* = 0.015), correlation coefficient −0.04 [95%CI: −0.12; 0.03], I^2^ = 0.0%] (Figure 2I). Egger’s test (t = −1.14, *p* = 0.46) indicated that there was no obvious publication bias for this relationship (Figure 2I).

## 4. Discussion

This study summarizes the studies that focused on individual associations between MetS risk factors and CAM among young people aged up to 19 years of age. The mean of the effect size of studies in the random-effect model obtained for all HRV indices with CMRF ranged from −0.12 to 0.13, and were significant at *p* = 0.001; they are considered small, based on Cohen criteria [33]. Our results are aligned with other study results [6,19,27,28]. However, van Biljon et al. [18] reported that rMSSD was correlated significantly with WC (r = −0.45) and with HDL (r = 0.42) among black South African Children (age = 11.85 ± 0.89 years) and falls under a moderate effect size [18]. It has been suggested that specific WC cut-off points for South African black people are needed for correct identification of metabolic conditions [34]. Also, black Africans exhibit a higher propensity towards insulin resistance, and a higher prevalence of hypertension and low HDL cholesterol levels, in contrast to lower rates of hypertriglyceridemia compared to Caucasians [35]. It has also been shown that for black South African diabetic men, cardiovascular disease risks were substantially increased with a WC >90 cm. The waist circumference cut off point of >94 cm has the potential to misclassify many black South African diabetic men at risk of cardiovascular diseases. The prevalence of hypertension, dyslipidemia, and insulin resistance were progressively greater among black South Africans at higher waist circumferences [36]. Farah et al. [16] reported that BMI was significantly correlated with HF and LF by −0.41, 0.46, respectively, and is classified under a moderate effect size [16]. This may be related to the population recruited, as 28.5% had abnormal CMRF, and 56.7% and 14.9% had 1 risk factor and 2 or more risk factors, respectively.

Pascol et al. [25] have proposed that obese children with decreased HDL, increased TGs and presence of higher cardiac sympathetic activity in the standing position (with decrease in SDNN, rMSSD, LF, LF:HF and increase in HF) and significant decrease in physical activity levels were among the key factors for future progression of cardiovascular diseases among children [25]. Stefanaki et al. [30] proposed an interesting finding that HRV indices (decrease in SDNN, rMSSD, HF and increase in mean of LF/HF ratio) were impaired in females irrespective of their glycemic status. They concluded that this may be due to stress disorders prevailing in females even from a young age, because they found no significant difference in body composition parameters between the pre-diabetic and euglycemic groups [30]. Another interesting study by Lee et al. [29] showed the effect of glucose regulation, BP and their combined effect on cardiac autonomic function in overweight obese 11–18 year old young people. They found no significant difference in the HRV parameters based on BP, glucose or interaction of both in overweight obese young people, even after adjustment of age and sex [29].

During growth in young age, hormonal fluctuations promote the growth of lean fat and bone mass, making youth more prone to insulin resistance [12]. Throughout adolescence, it seems to play a pivotal, reciprocal role in influencing changes in body composition [12]. Numerous studies have found that there is a correlation between the hypothalamic–pituitary–adrenal (HPA) axis hyperactivity, weight gain, and fat storage [37,38]. A hyperactive stress system may thus contribute to fat gain, particularly through a rise in HPA axis activity. On the flip side, increased fat mass seems to be a chronic pro-inflammatory, stressful state that affects the HPA axis as well. It is also thought that the presence of subclinical inflammation in obesity contributes to the disruption of metabolic equilibrium, suggesting that pro-inflammatory cytokines secreted by the adipocytes can play a potentially important pathogenic part [39]. In young people, whether they are released by adipocytes or they are induced by stress, pro-inflammatory molecules contribute to body composition disorders and adverse health outcomes in adulthood [40]. The young people are in the sensitive period of their physiological and psychological development, which means they are not able to deal with learning stress, sleep deprivation, interpersonal tension, and other external pressures and problems; thus, they are more likely to become depressed [13,14]. This may be the very important reason of neurovisceral regulation that may predict future cardiovascular risk among young people. It has already been shown that young people with depression also show impairment of autonomic nervous system functions, high levels of stress, and have slow fatigue recovery. Researchers have proposed that there are numerous metabolic pathways disrupted in young people with depression, including coenzyme Q biosynthesis, steroid metabolism, tyrosine metabolism, glycine-serine-threonine metabolism and pyrimidine metabolism [41].

The physiological effects of Coenzyme Q include reducing free radical production in the myocardium and muscle, and improving physical performance in people with heart disease [41,42]. A study found that the relative level of 4-hydroxyphenyl lactic acid, the key metabolite in the pathway of coenzyme Q biosynthesis, was significantly decreased in the depression group, indicating that metabolic deficiencies may contribute to depression [41]. Corticosterone is a key substance in the steroid hormone biosynthesis pathway and has been associated with obesity and metabolic disorders [43]. Previous studies have suggested that corticosterone levels are negatively related to depression severity [41,44]. Also, according to LC-MS studies, patients with depression exhibit differential plasma metabolites primarily associated with lipid metabolism, (such as LDL, unsaturated lipids, and cholesterol), amino acid metabolic pathways (such as alanine, taurine and glycine) and energy metabolic pathways (such as glucose, lactic acid and pyruvate) [41]. The di-hydro thymine biomarker is often used to assess the amount of thymine present in the body [45]. Thymine is an important physiological base unit of DNA and RNA. Research suggests that the rate of thymine metabolism is lower in depressed students than in healthy students, which could be due to insufficient sleep leading to myocardial fatigue. Dysregulation of stress reactivity may represent a mechanism by which psychological stress contributes to the development of future health and diseases [38]. It is possible that impaired sympathetic tone could be caused by increased central sympathetic tone or increased cardiovascular responsiveness to autonomic regulation, and may contribute to increased cardiovascular risk observed in patients [46].

Therefore, it can be deduced that even during the onset of MetS or prior to confirmation of a MetS state, we may screen young people at risk by monitoring their HRV to commence early prevention of future morbidities and mortalities in an efficient cost-effective way without the use of invasive methodologies.

### 4.1. SDNN and LF Associated Positively with HDL

The SDNN index may indicate overall variability. SDNN has been found to be significantly positively associated with HDL. Significant positive associations of HDL with SDNN were consistent with the results of three data sets [17,18]. On the contrary, Leppänen et al. [19] and Zhou et al. [20] reported that there was no significant association between HDL and SDNN [19,20]. Children aged between 5–6 years were only included in the study by Leppänen et al. and this may be the reason for a non-significant result [19]. Variations in the results presented by Zhou et al. [20] might be related to the fact that they completed the association study using male subjects only [20].

We found that HDL has been reported to correlate with HRV indices only in pre-pubertal age groups [18,28,47], and no association has been reported in children [6,19] and adolescents [17,27]. However, maturity parameters were not taken into consideration for most of the studies. The role of the maturational process is noteworthy in analyzed outcomes [48]. Also, interaction between prematurity and genetics/behavioral factors on HRV is still unclear. Paschol et al. [25] reported increased sympathetic activity to be negatively associated with HDL, but was not correlated to total cholesterol or LDL in Brazilian obese children (age = 9 to 11 years) [25]. Cardiovascular diseases are the primary cause of mortality worldwide [49]. The root pathophysiological mechanisms behind these diseases is atherosclerosis that starts developing during the early years of life [50,51]. Lipid-modifying therapy has been shown to prevent the progression of coronary atherosclerosis, confirming that abnormalities in plasma lipid concentrations are strongly associated with the progression of coronary artery disease and the occurrence of adverse clinical events [52]. Huikuri et al. [53] studied heart rate and its variability, measured by ambulatory electrocardiogram, and the angiographic progression of coronary artery disease in patients with reduced HDL cholesterol concentrations. In their multiple regression analysis including HRV, minimum heart rate, demographic and clinical variables, smoking, blood pressure, glucose, lipid measurements and lipid-modifying therapy, progression of focal coronary atherosclerosis were independently predicted by SDNN (beta = 0.24; *p* = 0.0001) [53]. Therefore, more studies focusing on SDNN measurements in younger populations are needed to explore further any relationships observed.

LF may be an indicator of baroreflex activity modulated by both the parasympathetic and sympathetic nervous systems. HDL was found to be significantly positively correlated with LF in young adults [54]. A study on young adults found that HDL was associated with high LF even after adjustment for T2D, depression and smoking [55]. Rodriguez-Colon et al.’s [56] results were similar to our results. This may be because the population included in their study was young adolescents with a mean age of 16.9 ± 2.24 years. However other studies did not report significant associations. This may be related to the fact that all studies had a mean age less than 16 years. A study including nine obese, dyslipidemic hypertensive and seven healthy normotensive individuals were studied. They reported a reduction in baroreflex activity, which was correlated with the rise in non-esterified fatty acids (r = −0.59, *p* = 0.02) but not with triglycerides [56].

### 4.2. rMSSD and HF Associated Negatively with TGs and WC

rMSSD and HF may reflect parasympathetic activity. Parasympathetic action helps in digestion and absorption of food by increasing the activity of the intestinal musculature, increasing gastric secretion and relaxing the pyloric sphincter. It is called the “rest and digest” division of the autonomic nervous system. Parasympathetic activity acts quickly in a matter of seconds. rMSSD captures fast changes in the instantaneous heart rate and reflects parasympathetic activity.

We found that TGs was negatively significantly associated with both indices of parasympathetic activity, including HF and rMSSD, and are consistent with Rodriguez-Colon et al.’s [17] findings among adolescents and Cayres et al.’s [28] findings among pre-pubertal adolescents. Interestingly, no studies have reported significant associations of HF with TGs among young people. A study reported that T2D patients with parasympathetic neuropathy had elevated fasting plasma C-peptide (*p* < 0.001) and TGs (*p* < 0.05) levels compared with patients without parasympathetic neuropathy. In addition, the age corrected E/I ratio correlated inversely with TGs (r = −0.31, *p* < 0.01) and fasting plasma C peptide in the T2D patients. Parasympathetic activity degrades with age. However, the findings of the study observed that most of the patients were younger who had parasympathetic neuropathy [57]. Parasympathetic neuropathy has been associated with coronary heart disease in T2D patients. Therefore, reduction in rMSSD and HF might be an indicator of high TGs levels with a need for screening for diabetes or pre-diabetes [58]. The link between hyperlipidemia and depressed HRV is not well studied. The possible hypothesis linking the association between TGs and HRV could be mediated by the role of catecholamines. Epinephrine and norepinephrine are not only known to increase heart rate but also increase lipolysis and free fatty-acid production, thereby increasing hepatic uptake. The blunting in the sympathetic response due to cardiac autonomic neuropathy may alter this pathway in addition to reducing heart rate. Hence, changes in the catecholamine response in cardiac autonomic neuropathy may be an important link explaining the association between these two clinical variables. Hypertriglyceridemia in patients with T2D is very hazardous and is negatively associated with HRV [59].

We found that WC was significantly associated with HF. WC has been correlated to parasympathetic activity in pre-pubertal adolescents [18,20], except in a study reported by Cayres et al. [28]. It is worth noting that only Cayres et al. [28] used maturation variables in their analysis for calculating associations between MetS risk factors and HRV indices [28]. Rodriguez-Colon et al. [17] have previously reported that an increase in one standard deviation in WC was significantly associated with lower HF, rMSSD and was also correlated with other HRV indices in children [17]. Associations between obesity and parasympathetic neuropathy is well established in non-diabetic [60] and T2D patients. A study reported that central adiposity and aging were associated with autonomic nervous system dysfunction in obese individuals. WC could be a marker of autonomic nervous system dysfunction in obese individuals without any MetS risk factors [61]. A study conducted on 159 participants (age = 29 to 96 years) reported that increasing WC was associated with decreasing SDNN and rMSSD in younger but not in older participants (*p* value for WC-by-age interaction  =  0.003) [62]. The literature shows that increased adipose tissue has been related to inferior autonomic modulation [63]. This pathway can be explained, at least partially, by the function of the sympathetic nervous system on the adrenal gland, stimulating the secretion of catecholamines: adrenaline and noradrenaline, which are responsible for lipolysis in adipose tissue via adrenoceptors β1 and β2 [64]. This pathway can be directly associated with triacylglycerol serum, as well as sympathetic hyperactivity stimulating the absorption of LDL in endothelial cells, contributing to atherosclerotic formation [64].

### 4.3. LF/HF Associated Positively with SBP and DBP

LF/HF may be an indicator of sympathovagal balance between sympathetic and vagal nerve activities [65]. SBP and DBP were found to be significantly positively correlated with LF/HF, consistent with Farah et al. [16]. Similar to Farah et al. another study among young people, with an average age above 19 years, reported that vagal inhibition plays an important role in addition to sympathetic activation in the alteration of sympathovagal imbalances in the genesis of prehypertension, especially in males. Gender and prehypertension status play an important role in the causation of sympathovagal imbalance. They suggested that the vagal tone in pre-hypertensives should be maintained at a higher level to prevent a further rise in blood pressure [66]. The reason for the consistent results with Farah et al. may be because their study participants were boys. Similarly, in this meta-analysis, the largest population included was also boys. BP was found to be significantly correlated with LF/HF in adolescents [17,27] and children [6,19], but not in pre-adolescents [18,28,47].

Tanaka et al. [67] demonstrated a significant correlation between resting arterial BP and Sympathovagal balance (*n* = 56, age = 13 to 16 years) among adolescents, but not among pre-pubertal adolescents (*n* = 71, age = 6 to 12 years) [67]. The results were not consistent; for instance, results from adolescents [16,27,28] and pre-adolescents [6] reported that sympathovagal balance is significantly correlated with BP. However, results among pre-adolescents in other studies [18,19] reported no association between BP and LF/HF. This is supported by the results of Tanaka et al. [67], and they suggested that BP levels may only be associated with cardiac autonomic nervous activity during puberty but not during pre-adolescence. However, our meta-analysis that combined the data from young people aged up to 19 years indicates that SBP and DBP are significantly positively correlated with LF/HF with a small effect. Our results specify that the association of BP with LF/HF is similar to that in adults, but that the association is low compared to adults. For instance, a study investigated the association of sympathovagal imbalance with CMRF in young subjects (age = 20.95 ± 2.56 to 21.28 ± 3.10 years), demonstrating an independent contribution of LF/HF ratio to prehypertension status (standardized β = 0.415, *p* < 0.001). The sympathovagal balance contributes to prehypertension status [68]. However, Lee et al. reported that there is no impact of high BP or pre-diabetes on HRV among overweight obese youth [29]. Therefore, we suggest that the relationship between BP and LF/HF still needs to be explored among adolescents.

As a matter of fact, HRV is not the only determinant of neurovisceral regulation: arterial pressure, respiration, stress and physical activity must be taken into consideration along with HRV. Normally, arterial blood pressure is controlled by altering parameters such as heart rate and stroke volume, arterial vascular resistance, and venous capacitance. Control of arterial blood pressure is carried out by both divisions of the autonomic nervous system. Parasympathetic stimulation affects only heart rate while sympathetic stimulation affects heart activity and blood vessel function. Increasing sympathetic nervous activity generally results in an increase in vascular tone as noradrenaline acts on vascular α-adrenoreceptors. Both β_2_- and α-adrenoreceptors are activated by circulatory adrenaline, causing respective vasodilation and vasoconstriction. As a negative feedback system, the arterial baroreceptor reflex controls arterial blood pressure under resting conditions [69]. Furthermore, respiratory sinus arrhythmia is characterized by heart rate variability in response to respiration. It is also accepted as a peripheral marker of cardiac-linked parasympathetic regulation, and as the index of emotion regulation [70]. Also, stress level and physical activity level must be considered along with HRV. As discussed, young people generally suffer from learning stress, sleep deprivation, interpersonal tension, and other external pressures and problems. It has already been shown that young people with depression also show impairment of autonomic nervous system functions, high levels of stress, and slow fatigue recovery leading to metabolic disturbances. Research also demonstrates that aerobic fitness enhances the integration of autonomic and cognitive functions [71]. Further, neurovisceral mechanisms are linked to emotion regulation, according to research. The results of a study show that yoga practitioners regulate their neurovisceral reactions differently from recreational athletes without yoga practice [72]. Therefore, we suggest that upcoming cardiovascular risk factors among young people can be easily altered by various functional factors or can be favorably reprogrammed by lifestyle modifications, including stress control and an increase in physical activity level, especially yoga or Tai chi that includes mind, body and breathing exercises. This study is an additional confirmation that functional elements, specifically indirect indexes of integrated regulation of bodily functions (HRV), are important in the modern approach to personalized-precision medicine, where prevention outperforms treatment among young population.

There are a few limitations in the present meta-analysis, including the results of heterogeneity (I^2^ > 50%). These were not further analyzed for subgroup analysis because of the limited numbers of included studies for each relationship. Also, we failed to investigate certain relationships such as associations between FGL and HRV indices or LDL and HRV indices because of the limited number of studies. Since only cross-sectional studies were selected for this review, the relationships proposed in this study do not establish a cause-effect relationship.

## 5. Conclusions

Lipid profile (HDL and TGs), WC and BP are associated with CAM in young people up to the age of 19 years. The use of HRV indices to predict future MetS risk factors including HDL, BP, WC and TGs in further studies related to young people (up to the age of 19 years) is recommended. However, due to the low number of association studies and the heterogeneity in the associated results of some of the variables such as BMI, FGL, LDL with HRV indices, more research is needed to explore their relationships with CAM.

## Data Availability

The extracted data used to support the findings of this study are available from the corresponding author upon request.

## Supplementary Materials

The following are available online at https://www.mdpi.com/article/10.3390/biology10080699/s1, Table S1: Synoptic table for heart rate variability indices, Table S2: Evaluation of methodological quality and risk of bias assessment and Table S3: Summary of the selected studies selected for systematic and meta-analysis.

## References

[1] Malpas S.C. (2010). Sympathetic Nervous System Overactivity and Its Role in the Development of Cardiovascular Disease. Physiol. Rev..

[2] Tsuji H., Larson M.G., Venditti F.J., Manders E.S., Evans J.C., Feldman C.L., Levy D. (1996). Impact of Reduced Heart Rate Variability on Risk for Cardiac Events. Circulation.

[3] Viljoen M., Claassen N. (2017). Allostatic load and heart rate variability as health risk indicators. Afr. Health Sci..

[4] Heart rate variability: Standards of measurement, physiological interpretation and clinical use (1996). Task Force of the European Society of Cardiology and the North American Society of Pacing and Electrophysiology. Circulation.

[5] Bond B., Cockcroft E.J., Williams C.A., Harris S., Gates P.E., Jackman S.R., Armstrong N., Barker A.R., Farah B.Q., Barros M.V.G. (2012). The Georgia Cardiovascular Twin Study: Influence of genetic predisposition and chronic stress on risk for cardiovascular disease and type 2 diabetes. Diabetes Care.

[6] Michels N., Matthys D., Thumann B., Marild S., De Henauw S. (2018). Children’s stress-related reports and stress biomarkers interact in their association with metabolic syndrome risk. Stress Health J. Int. Soc. Investig. Stress.

[7] Huang P.L. (2009). A comprehensive definition for metabolic syndrome. Dis. Model. Mech..

[8] Magge S.N., Goodman E., Armstrong S.C. (2017). The Metabolic Syndrome in Children and Adolescents: Shifting the Focus to Cardiometabolic Risk Factor Clustering. Pediatrics.

[9] Laitinen T., Hartikainen J., Niskanen L., Geelen G., Länsimies E. (1999). Sympathovagal balance is major determinant of short-term blood pressure variability in healthy subjects. Am. J. Physiol. Circ. Physiol..

[10] Rodríguez-Colón S.M., Bixler E.O., Li X., Vgontzas A.N., Liao D. (2011). Obesity is associated with impaired cardiac autonomic modulation in children. Int. J. Pediatr. Obes..

[11] Kaufman C.L., Kaiser D.R., Steinberger J., Kelly A.S., Dengel D.R. (2007). Relationships of Cardiac Autonomic Function with Metabolic Abnormalities in Childhood Obesity. Obesity.

[12] Stefanaki C. (2016). Prediabetes and Adolescence—Trends, Causes, Effects, and Screening. Endocrinology.

[13] Bufferd S.J., Dougherty L.R., Carlson G.A., Rose S., Klein D.N. (2012). Psychiatric Disorders in Preschoolers: Continuity from Ages 3 to 6. Am. J. Psychiatry.

[14] Merikangas K.R., Zhang H., Avenevoli S., Acharyya S., Neuenschwander M., Angst J. (2003). Longitudinal Trajectories of Depression and Anxiety in a Prospective Community Study. Arch. Gen. Psychiatry.

[15] Nanayakkara N., Curtis A.J., Heritier S., Gadowski A.M., Pavkov M.E., Kenealy T., Owens D.R., Thomas R.L., Song S., Wong J. (2021). Impact of age at type 2 diabetes mellitus diagnosis on mortality and vascular complications: Systematic review and meta-analyses. Diabetologia.

[16] Farah B.Q., Barros M.V.G., Balagopal B., Ritti-Dias R.M. (2014). Heart rate variability and cardiovascular risk factors in adolescent boys. J. Pediatr..

[17] Rodriguez-Colon S.M., He F., Bixler E.O., Fernandez-Mendoza J., Vgontzas A.N., Calhoun S., Zheng Z.J., Liao D., Rodríguez-Colón S.M., He F. (2015). Metabolic syndrome burden in apparently healthy adolescents is adversely associated with cardiac autonomic modulation-Penn State Children. Cohort. Metab. Exp..

[18] van Biljon A., McKune A.J., DuBose K.D., Kolanisi U., Semple S.J. (2019). Cardiac autonomic function and its association with cardiometabolic disease risk factors in Black South African children Auton. Neurosci. Basi. Clin..

[19] Leppänen M.H., Haapala E.A., Veijalainen A., Seppälä S., Oliveira R.S., Lintu N., Laitinen T., Tarvainen M.P., Lakka T.A. (2020). Associations of cardiometabolic risk factors with heart rate variability in 6- to 8-year-old children: The PANIC Study. Pediatr. Diabetes.

[20] Zhou Y., Xie G., Wang J., Yang S. (2012). Cardiovascular risk factors significantly correlate with autonomic nervous system activity in children. Can. J. Cardiol..

[21] The Joanna Briggs Institute (JBI) Checklist for Prevalence Studies: The Joanna Briggs Institute Critical Appraisal Tools for Use in JBI Systematic Reviews. Australia 2016, 3. https://jbi.global/critical-appraisal-tools.

[22] Kasten A.P., da Rosa B.N., Schmit E.F.D., Noll M., Candotti C.T. (2017). Prevalence of postural deviations in the spine in schoolchildren: A systematic review with meta-analysis. J. Hum. Growth Dev..

[23] Peterson R.A., Brown S.P. (2005). On the Use of Beta Coefficients in Meta-Analysis. J. Appl. Psychol..

[24] Suurmond R., van Rhee H., Hak T. (2017). Introduction, comparison, and validation of Meta-Essentials: A free and simple tool for meta-analysis. Res. Synth. Methods.

[25] Paschoal M.A., Trevizan P.F., Scodeler N.F. (2009). Heart rate variability, blood lipids and physical capacity of obese and non-obese children. Arq. Bras. Cardiol..

[26] Farah B.Q., Christofaro D.G.D., Cavalcante B.R., Andrade-Lima A., Germano-Soares A.H., Vanderlei L.C.M., Lanza F.C., Ritti-Dias R.M. (2018). Cutoffs of Short-Term Heart Rate Variability Parameters in Brazilian Adolescents Male. Pediatr. Cardiol..

[27] Soares A.H.G., Farah B.Q., Cucato G.G., Bastos-Filho C.J.A., Christofaro D.G.D., Vanderlei L.C.M., de Andrade Lima A.H.R., Ritti-Dias R.M., Germano Soares A.H., Farah B.Q. (2016). Is the algorithm used to process heart rate variability data clinically relevant? Analysis in male adolescents. Einstein.

[28] Cayres S.U., Vanderlei L.C.M., Silva D.R.P., Lima M.C.S., Barbosa M.F., Fernandes R.A. (2016). Cardiovascular and metabolic risk markers are related to parasympathetic indices in pre-pubertal adolescents. Cardiol. Young.

[29] Lee S., Cowan P.A., Wetzel G.T., Velasquez-Mieyer P. (2011). Prediabetes and blood pressure effects on heart rate variability, QT-interval duration, and left ventricular hypertrophy in overweight-obese adolescents. J. Pediatr. Nurs..

[30] Stefanaki C., Michos A., Latsios G., Tousoulis D., Peppa M., Zosi P., Boschiero D., Bacopoulou F. (2020). Sexual Dimorphism of Heart Rate Variability in Adolescence: A Case-Control Study on Depression, Anxiety, Stress Levels, Body Composition, and Heart Rate Variability in Adolescents with Impaired Fasting Glucose. Int. J. Environ. Res. Public Health.

[31] Plaza-Florido A., Alcantara J.M.A., Amaro-Gahete F.J., Sacha J., Ortega F.B. (2021). Cardiovascular Risk Factors and Heart Rate Variability: Impact of the Level of the Threshold-Based Artefact Correction Used to Process the Heart Rate Variability Signal. J. Med. Syst..

[32] Vrijkotte T.G.M., van den Born B.J.H., Hoekstra C.M.C.A., Gademan M.G.J., van Eijsden M., de Rooij S.R., Twickler M.T.B. (2015). Cardiac Autonomic Nervous System Activation and Metabolic Profile in Young Children: The ABCD Study. PLoS ONE.

[33] Cohen J. (1992). A power primer. Psychol. Bull..

[34] Owolabi E.O., Ter Goon D., Adeniyi O.V., Ajayi A.I. (2018). Optimal waist circumference cut-off points for predicting metabolic syndrome among low-income black South African adults. BMC Res. Notes.

[35] Gaillard T., Schuster D., Osei K. (2010). Differential impact of serum glucose, triglycerides, and high-density lipoprotein cholesterol on cardiovascular risk factor burden in nondiabetic, obese African American women: Implications for the prevalence of metabolic syndrome. Metabolism.

[36] Kalk W.J., Joffe B.I., Sumner A.E. (2011). The Waist Circumference of Risk in Black South African Men Is Lower Than in Men of European Ancestry. Metab. Syndr. Relat. Disord..

[37] Lucassen E.A., Cizza G. (2012). The Hypothalamic-Pituitary-Adrenal Axis, Obesity, and Chronic Stress Exposure: Sleep and the HPA Axis in Obesity. Curr. Obes. Rep..

[38] Turner A.I., Smyth N., Hall S.J., Torres S.J., Hussein M., Jayasinghe S.U., Ball K., Clow A.J. (2020). Psychological stress reactivity and future health and disease outcomes: A systematic review of prospective evidence. Psychoneuroendocrinology.

[39] Kyrou I., Chrousos G.P., Tsigos C. (2006). Stress, Visceral Obesity, and Metabolic Complications. Ann. N. Y. Acad. Sci..

[40] Stefanaki C., Pervanidou P., Boschiero D., Chrousos G.P. (2018). Chronic stress and body composition disorders: Implications for health and disease. Hormones.

[41] Zhao S., Chi A., Yan J., Yao C. (2020). Feature of Heart Rate Variability and Metabolic Mechanism in Female College Students with Depression. Biomed. Res. Int..

[42] Deichmann R.E., Lavie C.J., Dornelles A.C. (2012). Impact of Coenzyme Q-10 on Parameters of Cardiorespiratory Fitness and Muscle Performance in Older Athletes Taking Statins. Phys. Sportsmed..

[43] Wang M. (2005). The role of glucocorticoid action in the pathophysiology of the Metabolic Syndrome. Nutr. Metab..

[44] Felger J.C., Haroon E., Woolwine B.J., Raison C.L., Miller A.H. (2016). Interferon-alpha-induced inflammation is associated with reduced glucocorticoid negative feedback sensitivity and depression in patients with hepatitis C virus. Physiol. Behav..

[45] Duley J.A., Ni M., Shannon C., Norris R.L., Sheffield L., Harris M., van Kuilenburg A.B.P., Mead S., Cameron A., Helsby N. (2016). Towards a test to predict 5-fluorouracil toxicity: Pharmacokinetic data for thymine and two sequential metabolites following oral thymine administration to healthy adult males. Eur. J. Pharm. Sci..

[46] Pan J.X., Xia J.J., Deng F.L., Liang W.W., Wu J., Yin B.M., Dong M.X., Chen J.J., Ye F., Wang H.Y. (2018). Diagnosis of major depressive disorder based on changes in multiple plasma neurotransmitters: A targeted metabolomics study. Transl. Psychiatry.

[47] Zhou Y., Xie G., Wang J., Yang S., Leppänen M.H., Haapala E.A., Veijalainen A., Seppälä S., Oliveira R.S., Lintu N. (2020). Heart rate variability and cardiovascular risk factors in adolescent boys. J. Pediatr..

[48] Ciccone M.M., Scicchitano P., Zito A., Gesualdo M., Sassara M., Calderoni G., Di Mauro F., Ladisa G., Di Mauro A., Laforgia N. (2011). Different functional cardiac characteristics observed in term/preterm neonates by echocardiography and tissue doppler imaging. Early Hum. Dev..

[49] World Health Organisation Global Status Report on Noncommunicable Diseases 2014. Presented at the 2015. https://www.who.int/nmh/publications/ncd-status-report-2014/en.

[50] Berenson G.S., Srinivasan S.R., Bao W., Newman W.P., Tracy R.E., Wattigney W.A. (1998). Association between Multiple Cardiovascular Risk Factors and Atherosclerosis in Children and Young Adults. N. Engl. J. Med..

[51] McGill H.C., McMahan C.A., Herderick E.E., Malcom G.T., Tracy R.E., Strong J.P. (2000). Origin of atherosclerosis in childhood and adolescence. Am. J. Clin. Nutr..

[52] Gotto A.M. (1995). Lipid Lowering, Regression, and Coronary Events. Circulation.

[53] Huikuri H.V., Jokinen V., Syvänne M., Nieminen M.S., Airaksinen K.E.J., Ikäheimo M.J., Koistinen J.M., Kauma H., Kesäniemi A.Y., Majahalme S. (1999). Heart Rate Variability and Progression of Coronary Atherosclerosis. Arterioscler. Thromb. Vasc. Biol..

[54] Lin S., Lee I.H., Tsai H.C., Chi M.H., Chang W.H., Chen P.S., Chen K.C., Yang Y.K. (2019). The association between plasma cholesterol and the effect of tryptophan depletion on heart rate variability. Kaohsiung J. Med. Sci..

[55] Assoumou H.G.N., Pichot V., Barthelemy J.C., Dauphinot V., Celle S., Gosse P., Kossovsky M., Gaspoz J.M., Roche F. (2010). Metabolic Syndrome and Short-Term and Long-Term Heart Rate Variability in Elderly Free of Clinical Cardiovascular Disease: The PROOF Study. Rejuvenation Res..

[56] Gadegbeku C.A., Dhandayuthapani A., Sadler Z.E., Egan B.M. (2002). Raising lipids acutely reduces baroreflex sensitivity. Am. J. Hypertens..

[57] (2017). 28TH International symposium on the autonomic nervous system. Clin. Auton. Res..

[58] Eriksson K.F., Nilsson H., Lindgärde F., Österlin S., Dahlin L.B., Lilja B., Rosén I., Sundkvist G. (1994). Diabetes Mellitus but not Impaired Glucose Tolerance is Associated with Dysfunction in Peripheral. Nerves. Diabet. Med..

[59] Balikai F., Deshpande N., Javali S., Shetty D., Benni J., Shindhe V., Jaalam K., Kapoor N. (2020). The relationship between serum triglyceride level and heart rate variability in type 2 diabetes mellitus patients of North Karnataka. J. Diabetol..

[60] Valensi P., Thi B.N., Lormeau B., Pariès J., Attali J.R. (1995). Cardiac autonomic function in obese patients. Int. J. Obes. Relat. Metab. Disord..

[61] Fidan-Yaylali G., Yaylali Y.T., Erdogan Ç., Can B., Senol H., Gedik-Topçu B., Topsakal S. (2016). The Association between Central Adiposity and Autonomic Dysfunction in Obesity. Med. Princ. Pract..

[62] Windham B.G., Fumagalli S., Ble A., Sollers J.J., Thayer J.F., Najjar S.S., Griswold M.E., Ferrucci L. (2012). The Relationship between Heart Rate Variability and Adiposity Differs for Central and Overall Adiposity. J. Obes..

[63] Altuncu M.E., Baspinar O., Keskin M. (2012). The use of short-term analysis of heart rate variability to assess autonomic function in obese children and its relationship with metabolic syndrome. Cardiol. J..

[64] Zouhal H., Lemoine-Morel S., Mathieu M.-E., Casazza G.A., Jabbour G. (2013). Catecholamines and Obesity: Effects of Exercise and Training. Sport. Med..

[65] Eckberg D.L. (1997). Sympathovagal Balance. Circulation.

[66] Pal G.K., Pal P., Lalitha V., Dutta T.K., Adithan C., Nanda N. (2013). Sympathovagal Imbalance in Young Prehypertensives: Importance of Male-Female Difference. Am. J. Med. Sci..

[67] Tanaka H., Borres M., Thulesius O., Tamai H., Ericson M.O., Lindblad L.-E. (2000). Blood pressure and cardiovascular autonomic function in healthy children and adolescents. J. Pediatr..

[68] Pal G.K., Adithan C., Ananthanarayanan P.H., Pal P., Nanda N., Durgadevi T., Lalitha V., Syamsunder A.N., Dutta T.K. (2013). Sympathovagal Imbalance Contributes to Prehypertension Status and Cardiovascular Risks Attributed by Insulin Resistance, Inflammation, Dyslipidemia and Oxidative Stress in First Degree Relatives of Type 2 Diabetics. PLoS ONE.

[69] Kirkman E., Sawdon M. (2010). Neurological and humoral control of blood pressure. Anaesth. Intensive Care Med..

[70] Tonhajzerova I., Mestanik M., Mestanikova A., Jurko A. (2016). Respiratory sinus arrhythmia as a non-invasive index of ′brain-heart′ interaction in stress. Indian J. Med. Res..

[71] Alderman B.L., Olson R.L. (2014). The relation of aerobic fitness to cognitive control and heart rate variability: A neurovisceral integration study. Biol. Psychol..

[72] Wadden K.P., Snow N.J., Sande P., Slawson S., Waller T., Boyd L.A. (2018). Yoga Practitioners Uniquely Activate the Superior Parietal Lobule and Supramarginal Gyrus During Emotion Regulation. Front. Integr. Neurosci..

