# Supplementary material for: Association between Metabolic Syndrome Components and Cardiac Autonomic Modulation among Children and Adolescents: A Systematic Review and Meta-Analysis
Source: Biology (Basel). 2021 Jul 22;10(8):699. doi: 10.3390/biology10080699 (PMC8389259; doi:10.3390/biology10080699)
Supplement: Supplementary file 1 [file biology-10-00699-s001.zip › biology-1285927-supplementary.pdf]

Supplementary Materials

Table S1. Synoptic table for heart rate variability indices.

S.No.	Indices	Full Form	Clinical meaning
1	SDNN	Standard deviation of the time between two successive intervals	This is a time-domain parameter of Heart Rate Variability (HRV) that indicates the median of the variability in heart rate.<br>Good heart health is characterized by a higher SDNN; this indicates that the autonomic nervous system is flexible and adaptable. Conversely, a low SDNN indicates low health and insufficient adaptability.
2	rMSSD	Mean square root of the sum of differences between mean time between two successive intervals	It is a time-domain parameter of HRV. Based on beat-to-beat variations in heart rate, it describes changes mediated vagally.<br>In general, higher rMSSD indicates good cardiovascular health and a more adaptable autonomic nervous system. However, a low rMSSD indicates poor health and a lack of adaptability.
3	LF	Low Frequency	LF is a frequency domain parameter of HRV. It is defined as a low-frequency respiratory band that falls in the range of 0.04 to 0.15 Hz and is measured for at least 2 minutes.<br>An elevated LF level is a result of exaggerated sympathetic activity and may lead to migraines. A reduction in LF causes: fatigue, lethargy, fatigue, and reduced energy
4	HF	High Frequency	HF is also a frequency domain parameter of HRV. HF refers to a high-frequency respiratory band, which lies between 0.15 and 0.40 Hz and is typically recorded over a 1-minute period of time.<br>Generally, HF usually increases when HRV increases. Reduction of HF leads to: chronic stress, aging, reduced electrical stability of heart, indigestion.
5	LF/HF	Low Frequency/High Frequency ratio	It is a measure of the ratio between the parasympathetic and sympathetic nervous systems, normally determined from 24 hours of recording.<br>Increased LF/HF is an indication of good cardiovascular health and shows a healthy autonomic nervous system. In contrast, low LF/HF is a sign of poor health and of an insufficient ability to adapt.

Table S2. Evaluation of methodological quality and risk of bias assessment

**(JBI critical Appraisal checklist for prevalence studies)**										
Study	1	2	3	4	5	6	7	8	9	Total Score
(Farah et al. 2014)	y	y	O	y	y	y	y	y	O	7
Cayres et al. 2016)	y	y	y	y	y	y	y	y	y	9
(van Biljon et al. 2019)	y	y	O	y	y	y	y	y	O	7

(Zhou et al. 2012)	y	y	O	y	y	y	y	y	O	7
(Soares et al. 2016)	y	y	O	y	y	y	y	y	O	7
(Leppänen et al. 2020)	y	y	O	y	y	y	y	y	y	8
(Rodriguez-Colon et al. 2015)	y	y	O	y	y	y	y	y	y	8
(Michels et al. 2018)	y	y	y	y	y	y	y	y	O	8
(Kaufman et al. 2007)	y	y	O	y	y	y	y	y	O	7

Responses to criteria: Y = yes; N = No, O = Obscure, NO = Not Applicable. 1. Was the sample representative of the target population? 2. Were the study participants adequately recruited? 3. Was the sample size adequate? 4. Have the individuals studied and the recruitment environment been described in detail? 5. Was the data analysis done with sufficient coverage of the identified sample? 6. Were objective standard criteria used to measure the condition? 7. Was the condition reliably measured? 8. Was the statistical analysis appropriate? 9. Was the response rate adequate, and if not, was the low response rate adequately managed?

Table S3. Summary of the selected studies selected for systematic and meta-analysis

	Parameters included in the systematic review							Parameters included into meta-analysis	
	Authors	Sample size	Selected place	Sex	Number of boys(ratio of boys to total subjects)	Assessed cardio metabolic risk factors indices	Assessed Heart rate variability indices	Assessed cardio metabolic risk factors and Heart rate variability indices	Study Design
1	(Farah et al. 2014)	1152	Brazil	boys	1152 (1.00)	BP, BMI,WC, ≥2 MetS risk factors	HF, LF, LF/HF, rMSSD, SDNN	BP, BMI, WC, ≥2 MetS risk factors and HF, LF, LF/HF, rMSSD, SDNN	Cross sectional
2	(Cayres et al. 2016)	99	Brazil	both	49 (0.49)	HDL, TGs, BP	HF, rMSSD	HDL, TGs, BP and HF, rMSSD	Cross sectional
3	(van Biljon et al. 2019)	34	South Africa	both	14 (0.41)	BP, BMI,WC, HDL, TGs, FGL	HF,LF, LF/HF, rMSSD, SDNN	BP, BMI,WC, HDL, TGs, FGL and HF, LF, LF/HF, rMSSD, SDNN	Cross sectional
4	(Zhou et al. 2012)	180	china	both	110 (0.61)	BP, BMI, WC, HDL, TGs, FGL, ≥2 MetS	HF,LF, LF/HF, rMSSD,	BP, BMI,WC, HDL, TGs,	Cross sectional

						risk factors	SDNN	FGL, ≥2 MetS risk factors and HF, LF, LF/HF, rMSSD, SDNN	
5	(Soares et al. 2016)	1152	Brazil	boys	1152 (1.00)	BP, BMI, WC	HF,LF, LF/HF	BP,BMI,WC and HF, LF, LF/HF	Cross sectional
6	(Leppänen et al. 2020)	443	Finland	both	232 (0.52)	BP, WC, HDL, TGs, FGL, ≥2 MetS risk factors	HF,LF, LF/HF, rMSSD, SDNN	BP, WC, HDL, TGs, FGL, ≥2 MetS risk factors and HF, LF, LF/HF, rMSSD, SDNN	Cross sectional
7	(Rodriguez-Colon et al. 2015)	421	Pennsylvania	both	199 (0.470)	WC, HDL, TGs, ≥2 MetS risk factors	HF,LF, LF/HF, rMSSD, SDNN	WC, HDL, TGs, ≥2 MetS risk factors and HF, LF, LF/HF, rMSSD, SDNN	Cross sectional
8	(Michels et al. 2018)	264	Belgium	both	127 (0.48)	WC, HDL, TGs, BP	LF/HF	WC, HDL, TGs, BP and LF/HF	Cross sectional
9	(Kaufman et al. 2007)	36	US	both	18 (0.50)	BP, WC, HDL, TGs, FGL, ≥2 MetS risk factors	HF, LF, LF/HF, rMSSD, SDNN	None	Cross sectional
10	(Paschoal, Trevizan, and Scodeler 2009)	30	Brazil	both	15 (0.50)	≥2 MetS risk factors	rMSSD, SDNN	None	Cross sectional
11	(Stefanaki et al. 2020)	29	Greece	both	13 (0.44)	BMI, FGL, ≥2 MetS risk factors	HF, LF, LF/HF, rMSSD, SDNN	None	Case control study
12	(Lee et al. 2011)	128	Tennessee	both	47 (0.36)	FGL, BP, BMI	HF, SDNN	None	Descriptive correlational design
13	(Farah et al. 2018)	1152	Brazil	boys	1152 (1.00)	BP, WC, ≥2 MetS risk factors	HF, LF, LF/HF, rMSSD, SDNN	None	Cross sectional
14	(Plaza-Florido et al. 2021)	107	Spain	both	61 (0.57)	BP, WC, HDL, LDL, TGs, FGL, ≥2 MetS risk factors	HRV score	None	Cross sectional
15	(Vrijkotte et	1540	Dutch	both	779	BP, HDL, TGs,	Heart rate	None	Cross

al. 2015)	(0.51)	FGL	sectional

BP is blood pressure, FGL is fasting blood glucose; HDL is high density lipoprotein; LDL is low density lipoprotein, TGs is Triglycerides, WC is waist circumference, BMI is body mass index, HF is High Frequency, LF is Low Frequency, LF/HF is ratio of Low frequency and high frequency, rMSSD is mean square root of the sum of differences between mean time between two successive intervals, SDNN is standard deviation of the time between two successive intervals, HRV is Heart rate variability
